# Fatigue correlates with the decrease in parasympathetic sinus modulation induced by a cognitive challenge

**DOI:** 10.1186/1744-9081-10-25

**Published:** 2014-07-28

**Authors:** Kei Mizuno, Kanako Tajima, Yasuyoshi Watanabe, Hirohiko Kuratsune

**Affiliations:** 1 Pathophysiological and Health Science Team, RIKEN Center for Life Science Technologies, 6-7-3 Minatojima-minamimachi, Chuo-ku, Kobe, Hyogo 650-0047, Japan; 2 Department of Medical Science on Fatigue, Osaka City University Graduate School of Medicine, 1-4-3 Asahimachi, Abeno-ku, Osaka City, Osaka 545-8585, Japan; 3 Osaka City University, Center for Health Science Innovation, 3-1 Ofuka-cho, Kita-ku, Osaka City, Osaka 530-0011, Japan; 4 Department of Physiology, Osaka City University Graduate School of Medicine, 1-4-3 Asahimachi, Abeno-ku, Osaka City, Osaka 545-8585, Japan; 5 Department of Health Science, Faculty of Health Science for Welfare, Kansai University of Welfare Sciences, 3-11-1 Asahigaoka, Kashihara, Osaka 582-0026, Japan; 6 Clinical Center for Fatigue Science, Osaka City University Hospital, 1-5-7 Asahimachi, Abeno-ku, Osaka City, Osaka 545-8586, Japan; 7 Department of Comparative Pathophysiology, Veterinary Medical Sciences, Graduate School of Agricultural and Life Science, The University of Tokyo, 1-1-1 Yayoi, Bunkyo-ku, Tokyo 113-8657, Japan

## Abstract

**Background:**

It is known that enhancement of sympathetic nerve activity based on a decrease in parasympathetic nerve activity is associated with fatigue induced by mental tasks lasting more than 30 min. However, to measure autonomic nerve function and assess fatigue levels in both clinical and industrial settings, shorter experimental durations and more sensitive measurement methods are needed. The aim of the present study was to establish an improved method for inducing fatigue and evaluating the association between it and autonomic nerve activity.

**Methods:**

Twenty-eight healthy female college students participated in the study. We used a *kana* pick-out test (KPT) as a brief verbal cognitive task and recorded electrocardiography (ECG) to measure autonomic nerve activity. The experimental design consisted of a 16-min period of ECG: A pre-task resting state with eyes open for 3 min and eyes closed for 3 min, the 4-min KPT, and a post-task resting state with eyes open for 3 min and eyes closed for 3 min.

**Results:**

Baseline fatigue sensation, measured by a visual analogue scale before the experiment, was associated with the decrease in parasympathetic sinus modulation, as indicated the by ratio of low-frequency component power (LF) to high-frequency component power (HF), during the KPT. The LF/HF ratio during the post-KPT rest with eyes open tended to be greater than the ratio during the KPT and correlated with fatigue sensation. Fatigue sensation was correlated negatively with log-transformed HF, which is an index of parasympathetic sinus modulation, during the post-KPT rest with eyes open.

**Conclusions:**

The methods described here are useful for assessing the association between fatigue sensation and autonomic nerve activity using a brief cognitive test in healthy females.

## Background

Fatigue, defined as a difficulty in initiating or sustaining voluntary activities [1], is experienced by many people during or after a prolonged period of activity. Large community surveys have reported that up to half of the general adult population report fatigue [2,3]. This is also true in Japan, where more than one third of the population report chronic fatigue [4]. Epidemiological studies have reported that the female/male ratio of people with chronic fatigue in the general population is approximately 2:1 [5,6], and Pikó et al. reported that fatigue level was higher in female university students than in male university students [7]. Acute fatigue is a normal phenomenon that disappears after a period of rest. By contrast, chronic fatigue is sometimes irreversible and compensatory mechanisms that are useful in reducing acute fatigue are not effective [8]. Chronic fatigue is caused by the prolonged accumulation of acute fatigue. Thus, in order to avoid chronic fatigue, it is important to develop objective measures of fatigue and effective strategies to recover from and avoid the accumulation of acute fatigue.

Alterations of autonomic functions caused by fatigue in healthy people have been measured by electrocardiogram (ECG) and accelerated plethysmography and can provide objective biomarkers of fatigue [4]. Decreased parasympathetic sinus modulation and increased sympathetic sinus modulation were induced in healthy volunteers following a 30-min series of fatigue-inducing mental tasks [9,10]. After a prolonged cognitive load for 8 h, corresponding to a normal work day, we found that sympathetic hyperactivity based on decreased parasympathetic sinus modulation was positively correlated with subjective fatigue level [11]. This suggests that enhancement of sympathetic sinus modulation is closely related to fatigue induced by lengthy mental tasks of 30 min to 8 h. However, to measure autonomic nerve function and fatigue levels, both in clinical and industrial settings, better experimental designs, including briefer mental tasks and more sensitive measures for detecting changes in sympathetico-vagal balance, are needed.

In previous studies, for fatigue-inducing and fatigue-evaluating tests, we have used 30 min or more of the 2-back test [9,10] and an advanced trail making test [9-11], both of which require working memory or selective attention, to investigate the relation between fatigue and autonomic nerve functions. The *kana* pick-out test (KPT) is a divided-attention test (dual task) that has also been used as a fatigue-inducing and fatigue-evaluating mental task [11-13]. The KPT demands parallel processing during a 4-min task. Participants must select a subset of letters contained within a story while reading the story for comprehension for 2 min, and must then answer 10 questions about the contents of the story for 2 min. In the present study, we used the KPT as a brief but difficult mental task.

**Figure 1:**
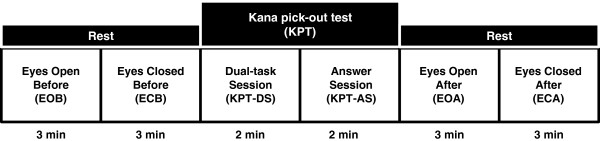
**Time course of the 16-min experimental period.** Experiment consists of performing the *kana* pick-out test (KPT) and resting states before and after the KPT.

Autonomic nerve function during mental fatigue was assessed during the KPT and while participants were sitting quietly with their eyes closed or open for few minutes rest before and after the KPT. We have previously recorded ECG and accelerated plethysmography with participants’ eyes open [14] or closed [10,11] during rest, but with eyes open only during the fatigue-inducing task. Attention levels are different between eyes open and eyes closed conditions, therefore the sympathetico-vagal balance is also considered to be different, suggesting that control of sympathetico-vagal balance could be evaluated by comparing these conditions [15]. Therefore, in the present study we measured ECG with both eyes closed and open to investigate the sensitivity of our measurement method for detecting changes in autonomic nerve activity produced by fatigue.The aim of the present study was to establish an improved fatigue measurement method for assessing the association between fatigue sensation and autonomic nerve activity in healthy female volunteers. We measured a 16-min period of ECG that covered a pre-KPT resting state with eyes open (3 min) and eyes closed (3 min), the 4-min KPT performed with eyes open, and a post-KPT resting state with eyes open (3 min) and eyes closed (3 min; Figure 1), and investigated the correlation between baseline fatigue sensation and alterations in autonomic nerve activity induced by the KPT.

## Methods

### Participants

The study group comprised 28 healthy female college students [age, 20.8 ± 0.5 years (mean ± SD)]. Individuals with a history of medical illness or taking chronic medications or supplemental vitamins were excluded. Current smokers were also excluded because smoking is closely associated with fatigue [16], sleep [17], and attentional task performance [18] and it has been reported that female cigarette smokers have abnormal sympathetic nerve activity [19]. These findings indicate that smoking affects fatigue and fatigue-related factors (sleep, task performance and autonomic nerve function). The study protocol was approved by the Ethics Committee of Kansai University of Welfare Sciences, and all participants provided written informed consent for participation in the study.

### Experimental design

The time schedule for the experiments is shown in Figure 1. On the day before the experiment and the day of the experimental, participants were instructed to avoid intensive physical and mental activities. We conducted the experiment for around 1 h within the period from 9:00 a.m. to noon in each participant. Before measurement of ECG, participants recorded their subjective sensation of fatigue on a visual analogue scale (VAS) from 0 (no fatigue) to 100 (complete exhaustion). To evaluate the autonomic nerve function during the resting state before the KPT, ECG was recorded while participants sat in a chair for 6 min: 3 min with their eyes open and 3 min with their eyes closed. Participants then performed the KPT for 4 min, and ECG was recorded throughout. ECG was again recorded during the resting state after the KPT: 3 min with eyes open and 3 min with eyes closed.

### Electrocardiogram

ECG was recorded continuously during the experiment using RF-ECG (GM3, Tokyo, Japan) and was analyzed using MemCalc/Bonaly Light (SuwaTrust/GMS, Tokyo, Japan). Frequency analyses for R-R interval variation were analyzed with the maximum entropy method, which is capable of estimating the power spectrum density from short time series data, and is adequate to examine changes in heart rate variability in different conditions of short duration [20,21]. The validity of the power spectral density of the maximum entropy method has been confirmed by comparing it to the autoregressive model [22]. The power spectrum resolution was 256 Hz. For the frequency analyses, the total power (TP) was calculated as the power within the frequency range 0 – 0.4 Hz. The very-low-frequency component power (VLF) was calculated as the power within the frequency range 0 – 0.05 Hz, the low-frequency component power (LF) was calculated as the power within the frequency range 0.04 – 0.15 Hz, and the high-frequency component power (HF) as the power within the frequency range 0.15 – 0.4 Hz. Absolute LF and HF values were transformed to normalized units (nu) as follows: [LF nu = LF/(TP – VLF) × 100] and [HF nu = HF/(TP – VLF) × 100] [23,24]. LF is simultaneously a marker of sympathetic sinus modulation increase and a marker of parasympathetic sinus modulation decrease, LF nu is a marker of parasympathetic sinus modulation decrease, HF nu is a marker of parasympathetic sinus modulation increase, and the LF/HF ratio expressed in nu is a marker of parasympathetic sinus modulation decrease [24].

### *Kana* pick-out test

The KPT requires parallel processing of reading and selection of letters, and also requires appropriate allocation of attentional resources to the two activities. Participants are shown a short story written in Japanese *kana* characters. They are required to find as many vowel symbols as possible within 2 min, while understanding the meaning of the story (dual-task session). Two min after the start of the test, they are asked 10 questions about the contents of the story over a 2 min period (answer session). Japanese *kana* characters consist of 66 phonetic symbols that include five vowels and the story consisted of 406 symbols with 61 vowels. The full score for this test is therefore 61, and the full comprehension score is 10.

### Statistical analyses

Log-transformed LF (ln LF), HF (ln HF) and LF/HF ratio (ln LF/HF ratio) in the conditions (pre-KPT rest with eyes open, pre-KPT rest with eyes closed, KPT dual-task session, KPT answer session, post-KPT rest with eyes open and post-KPT rest with eyes closed) were analyzed using [11,23] parametric one-way repeated measures analysis of variance (ANOVA). When statistically significant effects were found, intergroup differences were evaluated using the Tukey's honestly significant difference test. Stepwise multiple regression analyses were calculated between the VAS score for fatigue and autonomic nerve function. All *p*-values were two-tailed, and *p*-values less than 0.05 were considered significant. These analyses were performed with the IBM SPSS 20.0 software package (SPSS Inc, Chicago, IL).

## Results

**Figure 2:**
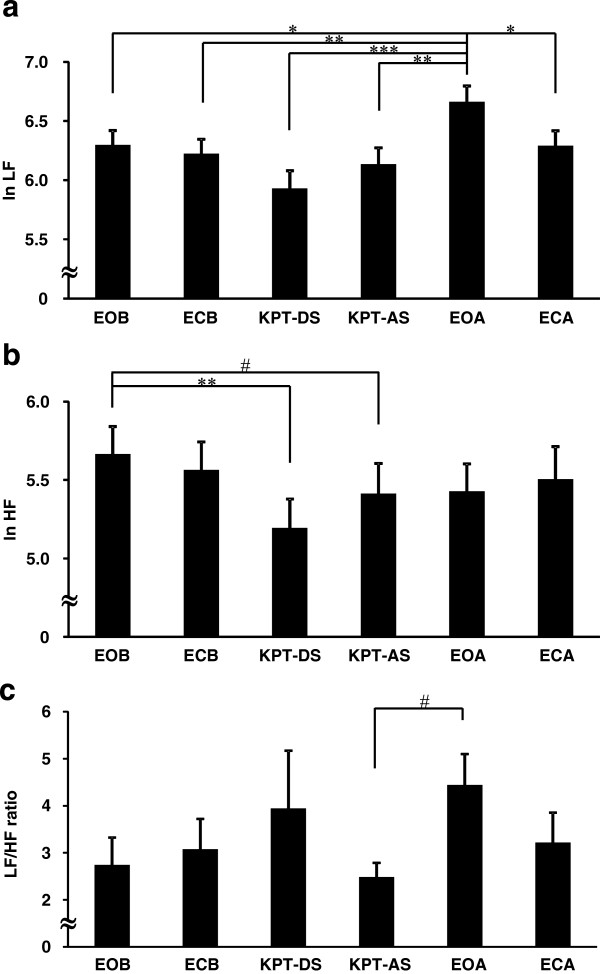
**Changes in autonomic nerve activity induced by the brief cognitive test. (a)** Log-transformed low-frequency component power (ln LF), **(b)** high-frequency component power (ln HF) and **(c)** the LF/HF ratio. EOB, eyes open; ECB, eyes closed before; KPT-DS, dual-task session of the *kana* pick-out test; KPT-AS, answer session of the *kana* pick-out test; EOA, eyes open after; ECA, eyes closed after. Values are presented as the mean and standard error of the mean. ^*^p < 0.05, ^**^p < 0.01, ^***^p < 0.001, significantly different from the corresponding values. ^#^*p* < 0.1, trend for a difference from the corresponding values.

The results for ECG are summarized in Figure 2. One-way repeated measures ANOVA revealed a significant main effect for ln LF [*F*(5, 27) = 8.46, *p* < 0.001] and ln HF [*F*(5, 27) = 3.16, *p* = 0.010], and a trend of a main effect for LF/HF ratio [*F*(5, 27) = 2.10, *p* = 0.069]. The ln LF of post-KPT rest with eyes open was significantly higher than that of pre-KPT rest with eyes open and closed, KPT dual-task session, KPT answer session, and post-KPT rest with eyes closed (Figure 2a). The ln HF of the dual-task session and the answer session were lower than that of pre-KPT rest with eyes open (Figure 2b). The LF/HF ratio of post-KPT rest with eyes open showed a trend to be greater than that of KPT answer session (Figure 2c).

**Figure 3:**
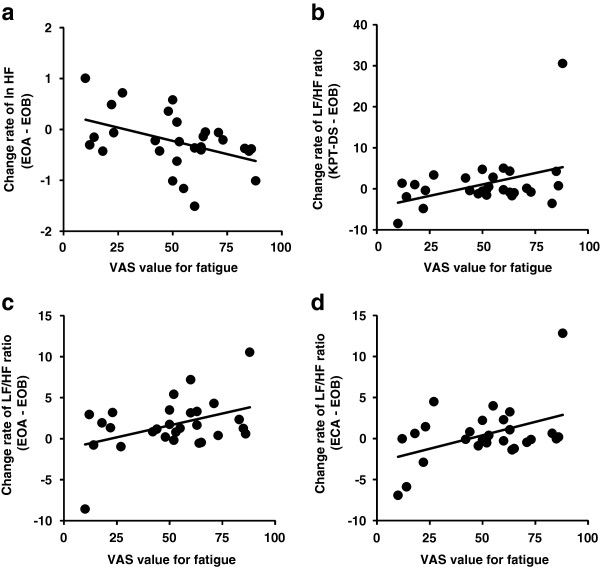
**Correlations between fatigue sensation and change in autonomic nerve activity during the short-duration cognitive test.** Correlations based on stepwise multiple regression analyses between baseline visual analogue scale (VAS) score for fatigue and **(a)** change of ln HF from EOB to EOA, **(b)** change of LF/HF ratio from EOB to KPT-DS, **(c)** change of LF/HF ratio from EOB to EOA, and **(d)** change of LF/HF ratio from EOB to ECA. EOB, eyes open before; KPT-DS, dual-task session of *kana* pick-out test; EOA, eyes open after; ECA, eyes closed after.

To investigate the effect of each participant’s subjective fatigue sensation on autonomic nerve activity, multiple regression analyses between baseline fatigue sensation and change in autonomic nerve activity from the pre-KPT rest with eyes open condition (baseline) to other conditions were performed. The change in each absolute power (ln TP, ln VLF, ln LF and ln HF) and derived power (LF nu, HF nu and LF/HF) was calculated by subtracting the value during pre-KPT rest with eyes open from the value during pre-KPT rest with eyes closed, KPT dual-task session, KPT answer session, post-KPT rest with eyes open, and post-KPT rest with eyes closed. Results of multiple regression analyses are shown in Figure 3. After stepwise selection in the absolute power variables, a significant negative relation was observed between VAS score for fatigue and change in ln HF in post-KPT rest with eyes open (β = −0.408, *p* = 0.043) based on a significant regression model (*p* = 0.043; Figure 3a). After stepwise selection in the derived power variables, there was also a positive correlation between VAS score for fatigue and change in LF/HF ratio in the KPT dual-task session (β = 0.397, *p* = 0.036; Figure 3b) and post-KPT rest with eyes open (β = 0.417, *p* = 0.027; Figure 3c) and post-KPT rest with eyes closed (β = 0.444, *p* = 0.018; Figure 3d) conditions based on each significant regression model (*p* < 0.05).

## Discussion

During the KPT dual-task session, there was a decrease in parasympathetic sinus modulation compared with pre-KPT rest with eyes open (Figure 2b) and fatigue sensation was associated with the decrease in parasympathetic sinus modulation and increase in sympathetic sinus modulation in this session (Figure 3b). This relation between the alteration of autonomic activity and fatigue sensation during the dual task may be related to interactions among the neural substrates of the KPT, fatigue and autonomic nerve function. We and another study group have used functional magnetic resonance imaging to show that the dorsolateral prefrontal cortex and cingulate cortex are activated during the KPT [25,26]. We have also used positron emission tomography to evaluate regional cerebral blood flow, and showed that the orbitofrontal cortex is associated with fatigue sensation assessed by VAS [27]. As for autonomic nerve function, a central autonomic network that controls sympathetico-vagal balance is comprised of the orbitofrontal cortex, medial prefrontal cortex, anterior cingulate cortex, insula, amygdala, bed nucleus of the stria terminalis, hypothalamus, periaqueductal gray matter, pons and medulla oblongata [28,29]. The anterior cingulate cortex plays a particularly crucial role in the central control of sympathetico-vagal balance [30]. There are anatomical and functional connections between the dorsolateral prefrontal cortex and medial prefrontal cortex, including the anterior cingulate cortex and the orbitofrontal cortex [31-34]. This indicates that there are interactions between the activities of task-dependent regions, fatigue sensation-related regions and autonomic nerve function-associated regions. Sympathoexcitatory subcortical threat circuits are normally under the inhibitory control of the medial prefrontal cortex [35-37]. During the KPT, wider prefrontal areas including the dorsolateral prefrontal cortex and part of the medial prefrontal cortex were more active in the single-task session than in the dual-task session [26]. More extension activation of prefrontal regions, which reflect mental effort, is also related to fatigue during a verbal working memory task [38]. These results suggest that fatigue which induces greater prefrontal activity corresponds to mental effort to accurately answer the questions and results in decreases in parasympathetic nerve activity and inhibitory capacity for sympathoexcitatory response.

In the present study, we focused on the difference in autonomic nerve activity between eyes open and eyes closed conditions. Previously, sympathetic hyperactivity was observed in the eyes closed condition after a fatigue-inducing task had been performed for 30 min [10] and 8 h [11]. Although sympathetic and parasympathetic sinus modulation were similar in pre-KPT rest with eyes open and pre-KPT rest with eyes closed, sympathetic nerve activity was higher and parasympathetic nerve activity was lower in post-KPT rest with eyes open than in post-KPT rest with eyes closed. Because attention level is different between eyes open and eyes closed conditions, sympathetic nerve activity is thought to be higher in eyes open condition than in the eyes closed condition [15]. However, before performing the KPT, the extent of the difference in sympathetic sinus modulation between eyes open and closed conditions was not observed because the brain network, including the prefrontal and anterior cingulate cortices, which play an important role in the regulation of autonomic nerve activity [39], was not driven; thus, control capacity by these brain regions was sufficient to inhibit the increase in sympathetic sinus modulation and decrease in parasympathetic sinus modulation in the eyes open condition. Because these brain regions are activated during the KPT [25,26], the increase in sinus sympathetic modulation and decrease in parasympathetic sinus modulation in the eyes open condition could not be adequately inhibited after the KPT. Inhibition of parasympathetic sinus modulation and the correlation between this activity and fatigue sensation was especially prevalent in this condition, suggesting that a brief mental task can be used to evaluate the change in autonomic nerve activity with fatigue if the eyes open condition is used. However, fatigue sensation was also associated with a decrease in parasympathetic sinus modulation in the post-KPT rest with eyes closed. Therefore, the extent to which parasympathetic nerve activity is inhibited in the recovery phase of the resting state in the eyes-closed condition may depend on the extent of fatigue.

Some researchers have found a difference in the fatigability of females and males with regard to physical and muscle fatigue [40,41]. In the case of cognitive fatigue, performance of the Stroop test under fatigue was lower in females than in male [42]. In the present study, we recruited only healthy females. This was to simplify and strengthen the analysis, and because epidemiological studies have shown that the number of females with chronic fatigue in the general population is twice that of males [5,6] and the fatigue level of female university students is higher than that of males [7]. In support of our findings, sympathetico-vagal balance was associated with fatigue in female volunteers aged from 19–24 years, but not in male volunteers of the same age [43].

Fatigue-related alterations of autonomic nerve activity have been reported in patients with chronic fatigue syndrome [44], multiple sclerosis [45,46], and primary biliary cirrhosis [47]. Here, we have shown that a 16-min period of ECG is adequate for evaluating the association between baseline fatigue sensation and altered autonomic nerve activity during and after a brief cognitive load. It is possible that this fatigue measurement method is useful to assess the severity of symptoms and treatment effects in these patients, and the measurement does not place excessive burden on patients.

### Limitations

There are limitations of this study. In order to generalize our results, further study involving a larger number of participants is essential. We did not follow the participants’ cycles of menstruation in the present study. Previous reports have reported an association between fatigue and menstruation [48,49], and future studies should consider participants’ menstruation cycles.

## Conclusions

Fatigue sensation was correlated with a decrease in parasympathetic sinus modulation during and after a cognitive test lasting 4 min, suggesting that we established a practical fatigue measurement method that can be used to assess the association between fatigue sensation and changes in autonomic nerve activity induced by brief cognitive testing. This newly developed method may contribute to evaluating the extent of physiological fatigue in not only healthy people, but also in those with fatigue-related disorders [50]. In addition, these methods also might contribute to investigations into the effect of interventions on recovery from fatigue via normalization of parasympathetic nerve alterations [51,52].

## Abbreviations

ANOVA: Analysis of variance; ECG: Electrocardiogram; HF: High-frequency component power; LF: Low-frequency component power; nu: Normalized unit; KPT: *Kana* pick-out test; TP: Total power; VAS: Visual analogue scale; VLF: Very low frequency component power.

## Competing interests

The authors declare that they have no competing interests.

## Authors’ contributions

KM took part in planning and designing the experiment and cognitive tests, performed the data analyses and drafted the manuscript. KT contributed to correct the data, and helped performing the data analyses. HK contributed to the design and planning of experiment, and corrected the data and helped draft the manuscript. YW took part in planning and designing the experiment and helped draft the manuscript. All authors read and approved the final manuscript.

## References

[B1] Chaudhuri A Behan PO Fatigue in neurological disorders Lancet 2004 363 9413 978 988 15043967 10.1016/S0140-6736(04)15794-2

[B2] Chen MK The epidemiology of self-perceived fatigue among adults Prev Med 1986 15 1 74 81 3714661 10.1016/0091-7435(86)90037-x

[B3] Pawlikowska T Chalder T Hirsch SR Wallace P Wright DJ Wessely SC Population based study of fatigue and psychological distress BMJ 1994 308 6931 763 766 7908238 10.1136/bmj.308.6931.763 PMC2539651

[B4] Watanabe Y Watanabe Y, Evengård B, Natelson BH, Jason LA, Kuratsune H Preface and mini-review: fatigue science for human health Fatigue Science for Human Health 2008 New York: Springer 5 11

[B5] Evengård B Jacks A Pedersen NL Sullivan PF The epidemiology of chronic fatigue in the Swedish Twin Registry Psychol Med 2005 35 9 1317 1326 16168154 10.1017/S0033291705005052

[B6] Van’t Leven M Zielhuis GA van der Meer JW Verbeek AL Bleijenberg G Fatigue and chronic fatigue syndrome-like complaints in the general population Eur J Public Health 2009 20 3 251 257 19689970 10.1093/eurpub/ckp113

[B7] Pikó B Barabás K Boda K Epidemiology of psychosomatic symptoms and its effect on the self-evaluation of general health in university students Orv Hetil 1995 136 31 1667 1671 (in Hungarian) 7637988

[B8] Beurskens AJ Bültmann U Kant I Vercoulen JH Bleijenberg G Swaen GM Fatigue among working people: validity of a questionnaire measure Occup Environ Med 2000 57 5 353 357 10769302 10.1136/oem.57.5.353 PMC1739950

[B9] Tanaka M Mizuno K Tajima S Sasabe T Watanabe Y Central nervous system fatigue alters autonomic nerve activity Life Sci 2009 84 7–8 235 239 19100749 10.1016/j.lfs.2008.12.004

[B10] Tanaka M Mizuno K Yamaguti K Kuratsune H Fujii A Baba H Matsuda K Nishimae A Takesaka T Watanabe Y Autonomic nervous alterations associated with daily level of fatigue Behav Brain Funct 2011 7 46 22032726 10.1186/1744-9081-7-46 PMC3214128

[B11] Mizuno K Tanaka M Yamaguti K Kajimoto O Kuratsune H Watanabe Y Mental fatigue caused by prolonged cognitive load associated with sympathetic hyperactivity Behav Brain Funct 2011 7 17 21605411 10.1186/1744-9081-7-17 PMC3113724

[B12] Mizuno K Tanaka M Fukuda S Imai-Matsumura K Watanabe Y Relationship between cognitive functions and prevalence of fatigue in elementary and junior high school students Brain Dev 2011 33 6 470 479 20846803 10.1016/j.braindev.2010.08.012

[B13] Tomoda A Mizuno K Murayama N Joudoi T Igasaki T Miyazaki M Miike T Event-related potentials in Japanese childhood chronic fatigue syndrome J Pediatr Neurol 2007 5 199 208

[B14] Tanaka M Shigihara Y Funakura M Kanai E Watanabe Y Fatigue-associated alterations of cognitive function and electroencephalographic power densities PLoS One 2012 7 4 e34774 22514666 10.1371/journal.pone.0034774 PMC3326030

[B15] Hori K Yamakawa M Tanaka N Murakami H Kaya M Hori S Influence of sound and light on heart rate variability J Hum Ergol 2005 34 1–2 25 34 17393762

[B16] Furlanetto KC Mantoani LC Bisca G Morita AA Zabatiero J Proenca M Kovelis D Pitta F Reduction of physical activity in daily life and its determinants in smokers without airflow obstruction Respirology 2014 19 3 369 375 24483840 10.1111/resp.12236

[B17] Hamidovic A De Wit H Sleep deprivation increases cigarette smoking Pharmacol Biochem Behav 2009 93 3 263 269 19133287 10.1016/j.pbb.2008.12.005 PMC2706278

[B18] Giessing C Thiel CM Alexander-Bloch AF Patel AX Bullmore ET Human brain functional network changes associated with enhanced and impaired attentional task performance J Neurosci 2013 33 14 5903 5914 23554472 10.1523/JNEUROSCI.4854-12.2013 PMC6618923

[B19] Middlekauff HR Park J Agrawal H Gornbein JA Abnormal sympathetic nerve activity in women exposed to cigarette smoke: a potential mechanism to explain increased cardiac risk Am J Physiol Heart Circ Physiol 2013 305 10 H1560 1567 23997107 10.1152/ajpheart.00502.2013 PMC4073979

[B20] Kanaya N Hirata N Kurosawa S Nakayama M Namiki A Differential effects of propofol and sevoflurane on heart rate variability Anesthesiology 2003 98 1 34 40 12502976 10.1097/00000542-200301000-00009

[B21] Takusagawa M Komori S Umetani K Ishihara T Sawanobori T Kohno I Sano S Yin D Ijiri H Tamura K Alterations of autonomic nervous activity in recurrence of variant angina Heart 1999 82 1 75 81 10377313 10.1136/hrt.82.1.75 PMC1729100

[B22] Sawada Y Ohtomo N Tanaka Y Tanaka G Yamakoshi K Terachi S Shimamoto K Nakagawa M Satoh S Kuroda S Iimura O New technique for time series analysis combining the maximum entropy method and non-linear least squares method: its value in heart rate variability analysis Med Biol Eng Comput 1997 35 4 318 322 9327605 10.1007/BF02534083

[B23] Task Force of the European Society of Cardiology (TFESC) & (North American Society of Pacing and Electrophysiology (NASPE) Heart rate variability: standards of measurement, physiological interpretation and clinical use Circulation 1996 93 1043 1065 8598068

[B24] Piccirillo G Ogawa M Song J Chong VJ Joung B Han S Magrì D Chen LS Lin SF Chen PS Power spectral analysis of heart rate variability and autonomic nervous system activity measured directly in healthy dogs and dogs with tachycardia-induced heart failure Heart Rhythm 2009 6 4 546 552 19324318 10.1016/j.hrthm.2009.01.006 PMC2756782

[B25] Mizuno K Tanaka M Tanabe HC Sadato N Watanabe Y The neural substrates associated with attentional resources and difficulty of concurrent processing of the two verbal tasks Neuropsychologia 2012 50 8 1998 2009 22571931 10.1016/j.neuropsychologia.2012.04.025

[B26] Tachibana A Noah JA Bronner S Ono Y Hirano Y Niwa M Watanabe K Onozuka M Activation of dorsolateral prefrontal cortex in a dual neuropsychological screening test: an fMRI approach Behav Brain Funct 2012 8 26 22640773 10.1186/1744-9081-8-26 PMC3464709

[B27] Tajima S Yamamoto S Tanaka M Kataoka Y Iwase M Yoshikawa E Okada H Onoe H Tsukada H Kuratsune H Ouchi Y Watanabe Y Medial orbitofrontal cortex is associated with fatigue sensation Neurol Res Int 2010 2010 671421 21188225 10.1155/2010/671421 PMC3003967

[B28] Benarroch EE Low PA The central autonomic network Clinical Autonomic Disorder 1997 2 Philadelphia: Lippincott-Raven 17 23

[B29] Loewy AD Loewy AD, Spyer KM Central autonomic pathways Central Regulation of Autonomic Functions 1990 New York: Oxford Univ Press 88 103

[B30] Critchley HD Mathias CJ Josephs O O'Doherty J Zanini S Dewar BK Cipolotti L Shallice T Dolan RJ Human cingulate cortex and autonomic control: converging neuroimaging and clinical evidence Brain 2003 126 Pt 10 2139 2152 12821513 10.1093/brain/awg216

[B31] Koski L Paus T Functional connectivity of the anterior cingulate cortex within the human frontal lobe: a brain-mapping meta-analysis Exp Brain Res 2000 133 1 55 65 10933210 10.1007/s002210000400

[B32] Paus T Castro-Alamancos MA Petrides M Cortico-cortical connectivity of the human mid-dorsolateral frontal cortex and its modulation by repetitive transcranial magnetic stimulation Eur J Neurosci 2001 14 8 1405 1411 11703468 10.1046/j.0953-816x.2001.01757.x

[B33] Petrides M Pandya DN Dorsolateral prefrontal cortex: comparative cytoarchitectonic analysis in the human and the macaque brain and corticocortical connection patterns Eur J Neurosci 1999 11 3 1011 1036 10103094 10.1046/j.1460-9568.1999.00518.x

[B34] Vogt BA Pandya DN Cingulate cortex of the rhesus monkey: II. Cortical afferents J Comp Neurol 1987 262 2 271 289 3624555 10.1002/cne.902620208

[B35] Amat J Baratta MV Paul E Bland ST Watkins LR Maier SF Medial prefrontal cortex determines how stressor controllability affects behavior and dorsal raphe nucleus Nat Neurosci 2005 8 3 365 371 15696163 10.1038/nn1399 PMC13675003

[B36] Thayer JF Sternberg E Beyond heart rate variability: vagal regulation of allostatic systems Ann N Y Acad Sci 2006 1088 361 372 17192580 10.1196/annals.1366.014

[B37] Thayer JF On the importance of inhibition: central and peripheral manifestations of nonlinear inhibitory processes in neural systems Dose Response 2006 4 1 2 21 18648636 10.2203/dose-response.004.01.002.Thayer PMC2477656

[B38] Lange G Steffener J Cook DB Bly BM Christodoulou C Liu WC Deluca J Natelson BH Objective evidence of cognitive complaints in chronic fatigue syndrome: a BOLD fMRI study of verbal working memory Neuroimage 2005 26 2 513 524 15907308 10.1016/j.neuroimage.2005.02.011

[B39] Tang YY Ma Y Fan Y Feng H Wang J Feng S Lu Q Hu B Lin Y Li J Zhang Y Wang Y Zhou L Fan M Central and autonomic nervous system interaction is altered by short-term meditation Proc Natl Acad Sci U S A 2009 106 22 8865 8870 19451642 10.1073/pnas.0904031106 PMC2690030

[B40] Hunter SK Sex differences in human fatigability: mechanisms and insight to physiological responses Acta Physiol (Oxf) 2014 210 4 768 789 24433272 10.1111/apha.12234 PMC4111134

[B41] Keller ML Pruse J Yoon T Schlinder-Delap B Harkins A Hunter SK Supraspinal fatigue is similar in men and women for a low-force fatiguing contraction Med Sci Sports Exerc 2011 43 10 1873 1883 21364478 10.1249/MSS.0b013e318216ebd4

[B42] Szinnai G Schachinger H Arnaud MJ Linder L Keller U Effect of water deprivation on cognitive-motor performance in healthy men and women Am J Physiol Regul Integr Comp Physiol 2005 289 1 R275 280 15845879 10.1152/ajpregu.00501.2004

[B43] Park YB Park YJ Ko YI Relationships of pulse waveform parameters to mood states and chronic fatigue J Altern Complement Med 2012 18 11 1050 1060 23072268 10.1089/acm.2011.0430

[B44] Newton JL Okonkwo O Sutcliffe K Seth A Shin J Jones DE Symptoms of autonomic dysfunction in chronic fatigue syndrome QJM 2007 100 8 519 526 17617647 10.1093/qjmed/hcm057

[B45] Keselbrener L Akselrod S Ahiron A Eldar M Barak Y Rotstein Z Is fatigue in patients with multiple sclerosis related to autonomic dysfunction? Clin Auton Res 2000 10 4 169 175 11029013 10.1007/BF02291352

[B46] Merkelbach S Dillmann U K?lmel C Holz I Muller M Cardiovascular autonomic dysregulation and fatigue in multiple sclerosis Mult Scler 2001 7 5 320 326 11724448 10.1177/135245850100700508

[B47] Newton JL Davidson A Kerr S Bhala N Pairman J Burt J Jones DE Autonomic dysfunction in primary biliary cirrhosis correlates with fatigue severity Eur J Gastroenterol Hepatol 2007 19 2 125 132 17272997 10.1097/01.meg.0000252629.96043.67

[B48] Negriff S Dorn LD Morningness/Eveningness and menstrual symptoms in adolescent females J Psychosom Res 2009 67 2 169 172 19616145 10.1016/j.jpsychores.2009.01.011 PMC4067163

[B49] Woods NF Most A Dery GK Prevalene of perimenstrual symptoms Am J Public Health 1982 72 11 1257 1264 6889817 10.2105/ajph.72.11.1257 PMC1650411

[B50] Goldstein DS Robertson D Esler M Straus SE Eisenhofer G Dysautonomias: clinical disorders of the autonomic nervous system Ann Intern Med 2002 137 9 753 763 12416949 10.7326/0003-4819-137-9-200211050-00011

[B51] Tajima K Tanaka M Mizuno K Okada N Rokushima K Watanabe Y Effects of bathing in micro-bubbles on recovery from moderate mental fatigue Ergon IJE HF 2008 30 2 134 145

[B52] Tanaka M Yamada H Nakamura T Watanabe Y Effects of pellet stove on recovery from mental fatigue Med Sci Monit 2012 18 3 CR148 153 22367125 10.12659/MSM.882519 PMC3560753

